# The role of the gut microbiota in the development of rheumatic diseases: a focus on fibromyalgia

**DOI:** 10.3389/fimmu.2026.1845199

**Published:** 2026-07-03

**Authors:** Yongli Zhao, Xingwen Xie

**Affiliations:** 1 Affiliated Hospital of Gansu University of Traditional Chinese Medicine, Lanzhou, China; 2 Gansu University of Traditional Chinese Medicine, Lanzhou, China

**Keywords:** central sensitization, dysbiosis, fibromyalgia, gut – brain axis, gut microbiota, rheumatic diseases

## Abstract

Fibromyalgia (FM) is a chronic widespread pain syndrome affecting 2%–4% of the population whose pathophysiology remains incompletely understood. Growing evidence implicates gut microbiota dysbiosis as a contributing factor, acting through immune, neuroendocrine, and metabolic pathways that may reinforce central sensitization. Consistent findings of reduced microbial diversity and altered metabolite profiles—including short-chain fatty acids, bile acids, and tryptophan derivatives—suggest mechanistic links between the gut and FM symptoms. Microbiota-targeted interventions such as probiotics, dietary modification, and fecal microbiota transplantation have shown preliminary benefits, though evidence remains limited by small sample sizes and methodological heterogeneity. This review synthesizes current knowledge on the role of the gut microbiota in FM within the broader context of rheumatic diseases and discusses future research directions.

## 1 Introduction

**Figure 1:**
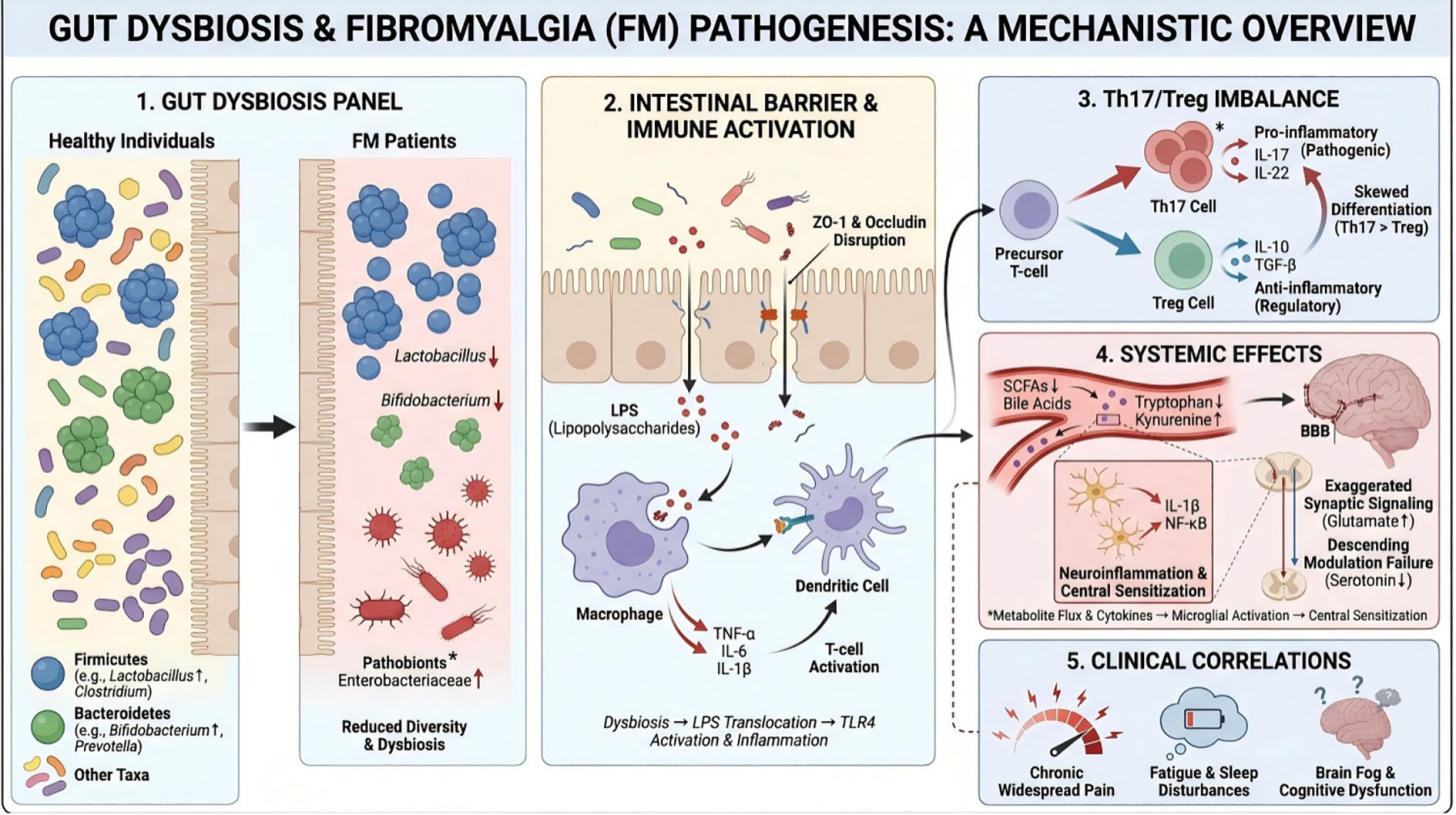
Proposed mechanisms by which gut microbiota dysbiosis contributes to FM pathogenesis. Dysbiosis leads to loss of beneficial bacteria and overgrowth of pathobionts, compromising intestinal barrier integrity and enabling translocation of microbial components (e.g., LPS) into circulation. This triggers TLR4-mediated innate immune activation, pro-inflammatory cytokine release (TNF-α, IL-6, IL-1β), and Th17/Treg imbalance. Concurrently, disrupted microbial metabolism reduces SCFAs and bile acids and perturbs the tryptophan-kynurenine pathway. These converging immune and metabolic signals act on the gut–brain axis to promote neuroinflammation, microglial activation, and central sensitization, collectively driving the characteristic symptoms of FM including chronic pain, fatigue, sleep disturbance, and cognitive dysfunction.

Fibromyalgia (FM) is a prevalent chronic pain syndrome among rheumatic diseases, but distinct from inflammatory rheumatic disorders (1–3). It is defined by widespread musculoskeletal pain and often features debilitating fatigue, nonrestorative sleep, and “brain fog, “ or cognitive impairment (4, 5). FM occurs in 2% to 4% of the general population and is three to four times more prevalent in women than men (6). Despite its significant disease burden, our understanding of the etiology and pathogenesis of FM is limited and, to date, diagnosis is based on clinical symptoms and the exclusion of other potential causes (7, 8). FM is currently thought to be a form of central sensitization syndrome, where the central nervous system displays an increased sensitivity to pain in the absence of peripheral inflammatory or autoimmune indicators. Earlier research has also implicated a variety of factors in FM, including dysfunction of the hypothalamic-pituitary-adrenal (HPA) axis, autonomic dysfunction, neuroimmune activation, and mitochondrial dysfunction (9). The pathology of FM is likely multifactorial, and no single mechanism can account for its development, suggesting that a holistic view of the disease is needed (10). Recently, the role of the gut microbiota in FM has provided an intriguing link to explore (11). It has been demonstrated that the gut microbiota in FM patients differs from that of healthy controls, and such changes may affect immune function, metabolic products, and neurochemicals, which in turn lead to pain and other symptoms (12). Given increasing evidence supporting the role of the gut microbiota in chronic diseases, including autoimmune rheumatic diseases, unraveling its role in FM may lead to both improved understanding of disease mechanisms and new tools for diagnosis and treatment. The proposed mechanisms of gut microbiota dysbiosis contributing to fibromyalgia are outlined in **Figure 1**.

## 2 Rheumatic diseases and the gut microbiota

Rheumatic conditions cover a wide range of conditions that affect the joints, muscles, connective tissues and immune system (13), such as rheumatoid arthritis (RA), systemic lupus erythematosus (SLE), ankylosing spondylitis (AS), psoriatic arthritis, and FM (14). Over the last 10 years, considerable evidence has emerged of a connection between dysbiosis of gut microbiota and several rheumatic diseases (15, 16). For instance, increased abundance of the gut bacterium Prevotella copri has been linked to the onset of autoimmune disease in RA (17). It has been speculated that dysbiosis increases the permeability and leakage of the gut mucosal barrier (also known as “leaky gut”), translocation of bacterial antigens, such as lipopolysaccharide (LPS), into the systemic circulation, and subsequent chronic inflammation (18). Dysbiosis can also affect immune homeostasis in the gut by shifting the balance between pro-inflammatory Th17 cells and anti-inflammatory regulatory T (Treg) cells, a feature of several rheumatic conditions, such as RA, AS, and SLE (19, 20). In SLE, overrepresentation of Ruminococcus gnavus has been linked to the translocation of nucleic acid antigens (21). Taken together, these findings suggest that the gut microbiota are involved in the development of traditional rheumatic diseases via a “gut-immune-joint axis” (22). Some researchers have also considered the possibility of a gut-joint axis, in which immune events in the gut can have remote effects on the joints and other organs, resulting in rheumatic disease. Therefore, manipulating the gut microbiota has become an attractive therapeutic target, with dietary modification, probiotic/prebiotic administration, and fecal microbiota transplantation having the capacity to improve immune dysregulation and disease activity (23).

### 2.1 Fibromyalgia and the gut microbiota

While FM has historically not been associated with autoimmunity, there is growing evidence that patients display subtle signs of systemic inflammation and immune activation, such as mild increases in circulating pro-inflammatory cytokines, and evidence of microglial activation (24–26). In fact, FM is often accompanied by functional bowel symptoms, and over half of patients with FM also meet criteria for irritable bowel syndrome (IBS), implying a potential gut-brain axis dysfunction (27). In 2019, the first report directly linking FM to the gut microbiota found that about 20 bacterial taxa were overrepresented or underrepresented in patients with FM compared to healthy subjects, in association with lower alpha diversity (28). While this was a correlational study and the first to report a consistent gut microbial signature in FM, it paved the way for further research (28).

Since 2020, several cohort studies have also provided confirmation of gut microbiota dysbiosis in FM (29, 30). A systematic review of 11 studies to date (up to 2023) involving 455 patients with FM and 385 controls has shown consistent gut microbiota dysbiosis, with lower microbial diversity, as measured by lower Shannon indices, along with changes in the gut microbiota composition (31).Reported abnormalities include an altered Bacteroidetes/Firmicutes ratio and aberrant abundance of specific taxa, such as members of the family Lachnospiraceae and the genus Prevotella (32). Although individual studies have not been entirely consistent regarding the specific taxa involved, the overall pattern of dysbiosis has been remarkably consistent. Importantly, these microbial alterations are often correlated with clinical manifestations: lower microbial diversity is associated with greater pain severity and fatigue, while the abundance of certain taxa correlates with anxiety and depressive symptoms (33, 34).

For example, a 2025 study by Lopez de Coca and colleagues reported a high prevalence of small intestinal bacterial overgrowth (SIBO) in patients with FM, approximately 59%, with methane-dominant overgrowth being particularly common and significantly more frequent than in controls (35). The same study found increased abundance of methanogenic archaea, such as Methanobrevibacter, along with reduced levels of beneficial butyrate-producing commensals, changes that may be related to gastrointestinal symptoms such as bloating and constipation (35)a 2023 study by the McGill University group in Canada identified abnormalities in the serum secondary bile acid profile (36). One secondary bile acid, α-muricholic acid, was present at only approximately one-fifth of the level observed in healthy controls and was negatively correlated with pain intensity and fatigue severity (36). On this basis, the authors proposed that certain gut microbial communities may influence chronic pain thresholds by modulating bile acid metabolism, thereby providing a novel perspective on FM pathogenesis (36). Taken together, evidence accumulated over the past five years strongly suggests a close relationship between FM and the gut microbiome, although the causal nature and precise mechanisms of this association remain to be fully elucidated (37).

## 3 Mechanisms by which the gut microbiota influences fibromyalgia: immune, neuroendocrine, and metabolic pathways

### 3.1 Immune pathways: gut microbiota and low-grade inflammation

The gut microbiota serves as a crucial educator of the host immune system through its continuous interaction with the mucosal immune network (38). Under physiological conditions, it helps maintain mucosal immune homeostasis, promotes the development of regulatory T (Treg) cells, and provides colonization resistance against pathogens (39, 40). However, this balance is disrupted under dysbiotic conditions. A large body of evidence indicates that dysbiosis increases intestinal permeability, allowing microbial-associated molecules such as LPS and peptidoglycan to enter the circulation (41). In FM, although overt peripheral inflammation is generally absent, modest elevations in markers such as LPS-binding protein suggest occult microbial translocation and immune activation. LPS can activate pattern-recognition receptors, including TLR4, thereby inducing monocytes and macrophages to release pro-inflammatory cytokines, including TNF-α, IL-1β, and IL-6 (42, 43). Although cytokine elevations in FM are less pronounced than those observed in autoimmune diseases such as RA, this state of low-grade systemic inflammation may nevertheless contribute to central sensitization and symptom persistence (44, 45).

**Figure 2:**
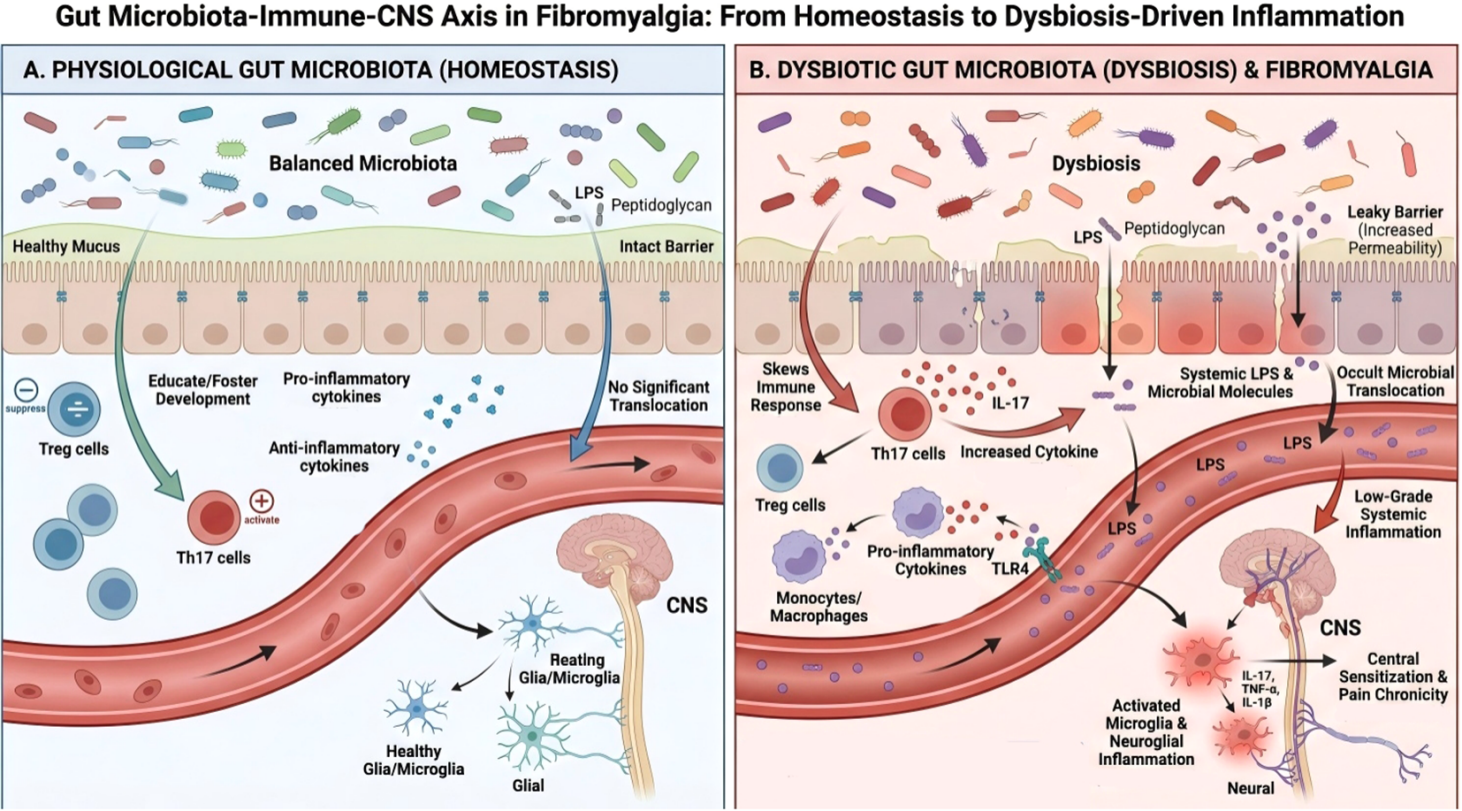
Illustrates the gut microbiota–immune–CNS axis in FM. **(A)** depicts homeostasis: a balanced microbiota supports intestinal barrier integrity, Th17/Treg equilibrium, and controlled cytokine production, maintaining normal microglial activity. **(B)** depicts dysbiosis: reduced diversity and pathobiont expansion impair barrier function, enabling translocation of LPS and peptidoglycan. TLR4 activation drives macrophage release of TNF-α, IL-1β, and IL-6, while Th17 polarization elevates IL-17. These signals propagate across the gut–brain axis, promoting microglial activation, neuroinflammation, and central sensitization underlying chronic widespread pain in FM.

Dysbiosis may also promote excessive activation of T helper 17 (Th17) cells while impairing Treg function, thereby disrupting the balance between pro-inflammatory and anti-inflammatory immune responses (46, 47). Cytokines produced by Th17 cells, particularly IL-17, may enhance neural excitability and exacerbate pain (48). In recent years, elevated markers of glial activation have also been detected in the cerebrospinal fluid of patients with FM, suggesting that neuroglial inflammation may amplify pain signaling (49, 50). Gut-derived inflammatory mediators may represent one trigger for such glial activation (51). Animal experiments have shown that administration of LPS to germ-free mice induces microglial activation and increases pain sensitivity (52). In the aforementioned mouse fecal microbiota transplantation model, colonization with FM-associated microbiota not only induced hyperalgesia but also increased spinal microglial activation and the proportion of peripheral classical monocytes, further supporting a link between microbially mediated immune activation and pain behavior (53–55). Thus, the gut microbiota may provide inflammatory “fuel” for the onset and progression of FM by promoting chronic low-grade inflammation and aberrant immune cell activation (56, 57). The immune mechanisms linking gut microbiota dysbiosis to neuroinflammation and pain in FM are summarized in **Figure 2**.

### 3.2 Neuroendocrine pathways: dysregulation of the gut–brain axis

The gut has often been described as a “second brain,” reflecting the bidirectional communication between the intestinal microbiota and the central nervous system through the gut–brain axis (58, 59). This communication operates through three principal pathways: neural signaling via the vagus nerve and enteric nervous system, endocrine signaling mediated by stress hormones such as cortisol, and immune signaling mediated by inflammatory cytokines (60, 61). Under conditions of stress, activation of the hypothalamic–pituitary–adrenal (HPA) axis leads to glucocorticoid release, which can alter intestinal barrier integrity and microbial composition (62, 63). Conversely, microbial metabolites and neuroactive compounds can act on vagal afferents or enteric neurons, thereby transmitting signals back to the brain (64). Patients with FM frequently exhibit HPA axis dysregulation, including abnormal diurnal cortisol rhythms and blunted cortisol responses to stress, as well as sympathetic overactivation, both of which are thought to be related to chronic stress and impaired pain modulation (65–67).

**Figure 3:**
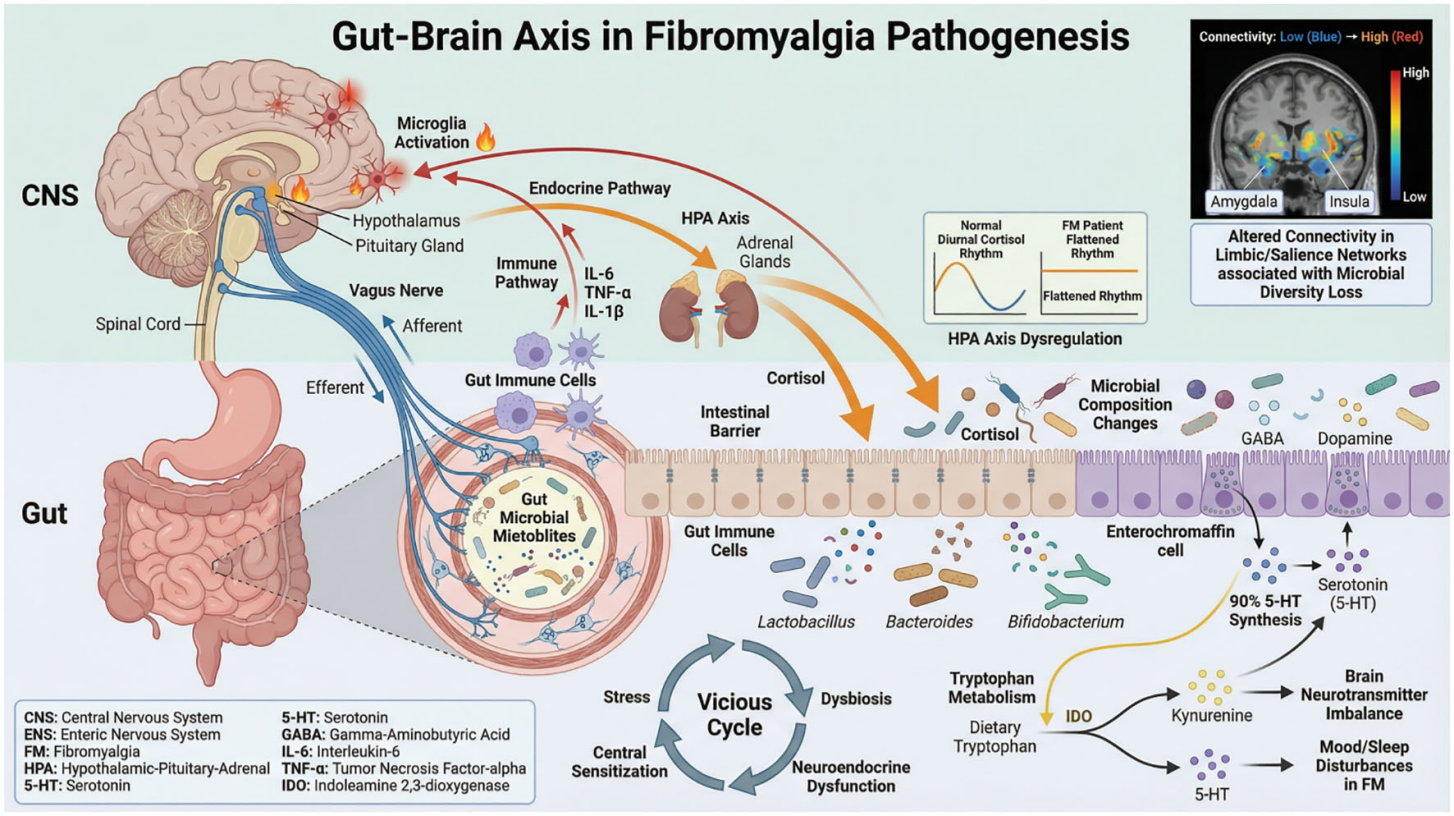
Illustrates the bidirectional gut–brain axis in FM. Dysbiosis disrupts the intestinal microecosystem, promoting cytokine release (IL-6, TNF-α, IL-1β) that enters systemic circulation and affects CNS function. Microbial neuroactive metabolites—including SCFAs, GABA, dopamine, and serotonin (5-HT)—communicate with the brain via vagal, endocrine, and immune routes. HPA axis dysregulation leads to altered cortisol secretion, while kynurenine pathway perturbations reduce serotonin availability, contributing to mood and sleep disturbances. Together these pathways establish a vicious cycle of stress, dysbiosis, neuroendocrine dysfunction, and central sensitization that sustains chronic pain and multisystem symptoms in FM.

In recent years, gut microbiota dysbiosis has been increasingly proposed as one of the factors contributing to this neuroendocrine imbalance (68). Deficiency of beneficial microbes and potential chronic intestinal infection may continuously stimulate the stress axis and increase sympathetic tone. In addition, gut microorganisms can produce a wide range of neuroactive compounds, including serotonin (5-HT), γ-aminobutyric acid (GABA), and catecholamines (69). Approximately 90% of serotonin is synthesized in the gut through interactions between the microbiota and enterochromaffin cells (70). In addition to its local role in regulating intestinal motility, serotonin may influence central emotional and pain pathways via the vagus nerve (71). The sleep disturbances and mood symptoms commonly observed in FM suggest dysregulation of central neurotransmitters such as 5-HT and norepinephrine, and the gut microbiota may contribute by modulating the metabolism and absorption of neurotransmitter precursors such as tryptophan (72–74). Notably, a 2024 magnetic resonance imaging (MRI) investigation associated changes in brain function among patients with FM with gut microbial makeup (75). Nhu and colleagues reported that the abundance of certain gut bacterial genera was significantly associated with functional connectivity within the salience network in women with FM, with Phascolarctobacterium abundance predicting depression severity and salience network connectivity (76, 77). Additionally, a reduction in microbial diversity was associated with more acute anxiety and depressive symptoms (78). These findings support the notion that the gut microbiota contributes to the neuropathophysiology of FM through neural pathways and stress-related mechanisms (79). In summary, the gut–brain axis may create a bidirectional feedback loop in FM: stress induces microbial dysbiosis, which in turn aggravates neuroendocrine imbalance and central sensitization, thereby establishing a vicious cycle (80). Disrupting this cycle may therefore represent a promising therapeutic goal (81). The bidirectional interactions between gut microbiota and the central nervous system in FM are summarized in **Figure 3**.

### 3.3 Metabolic pathways: microbial metabolites and pain modulation

The gut microbiota is a major metabolic organ that produces many metabolites with diverse physiological effects (82). In relation to FM, particular attention has been given to several classes of microbial metabolites (83). The first are short-chain fatty acids (SCFAs) such as acetate, propionate, and butyrate, generated by fermentation of dietary fiber by anaerobic gut bacteria (84). SCFAs regulate immune responses through G protein-coupled receptors (FFAR2, FFAR3) and may also affect hypothalamic and microglial activities following transport across the blood–brain barrier (85, 86). Deficiency of butyrate-producing bacteria is a recognized feature of dysbiosis (87). Some FM studies have reported associations between reduced stool SCFA concentrations and greater fatigue or pain severity; however, other studies have failed to detect significant SCFA differences between FM patients and healthy controls, and at least one investigation reported elevated rather than reduced propionate levels in FM patients (88–90). This inconsistency may reflect differences in dietary composition, stool collection methods, and analytical platforms across studies. It has been hypothesized that low butyrate may compromise intestinal epithelial barrier function and anti-inflammatory signaling, while altered propionate levels might influence pain thresholds via vagal afferents, but these mechanistic proposals remain to be experimentally validated in FM-specific models (36, 91, 92).

The second group are bile acids. As mentioned above, Minerbi and colleagues found that patients with FM had substantial disruption of secondary bile acids (93). Bile acids play a role in digestion and also in metabolism and inflammation as signaling molecules for farnesoid X receptor (FXR) and TGR5 (94, 95). Given that TGR5 is found in sensory neurons and immune cells, the dramatic decrease in α-muricholic acid suggests that altered activity of specific bile acids may contribute to pain in FM (96). In mice that received FMT and developed fibromyalgia-like hyperalgesia, administration of a bile acid analogue decreased pain, suggesting an indirect role of the bile acid pathway in microbiota-induced pain (97).

A third mechanism is tryptophan metabolism. Microbes in the gut metabolize tryptophan to produce indoles and kynurenines, which modulate the tryptophan-kynurenine pathway and serotonin in the host (98). Depression and insomnia are prevalent in FM patients, symptoms that have been linked to low serotonin (99). Some have suggested that the overgrowth of Bacteroides may use excess tryptophan for microbial metabolism, thus reducing the supply of tryptophan for host serotonin synthesis, or conversely, enhance the accumulation of kynurenine catabolites that mediate fatigue (98). While this theory is yet to be comprehensively tested in FM, preliminary evidence has been reported in other conditions including chronic fatigue syndrome (100, 101).

Finally, metabolomics has provided additional insight into metabolic abnormalities in FM. A 2024 metabolomic study combined with machine learning identified disruptions in tyrosine, purine, pyrimidine, and glutamine metabolism in the blood of patients with FM, and these disturbances were closely associated with fatigue symptoms (102). Some of these metabolic alterations may directly or indirectly result from changes in microbial function. Overall, the gut microbiota can remotely modulate host immune and neural function through its metabolic products (103). In FM, disruption of microbial metabolism may therefore represent a key mechanism underlying symptom initiation or exacerbation (104). For example, microbiota-induced reductions in SCFAs and specific bile acids may weaken anti-inflammatory and analgesic mechanisms, whereas disturbances in tryptophan metabolism and other metabolic pathways may promote pain sensitivity and fatigue (105, 106). Targeting microbial metabolic pathways may therefore provide a new entry point for understanding and treating FM (107).

## 4 The role of the gut microbiota in fibromyalgia

### 4.1 Animal model studies: clues to causality

To determine whether the gut microbiota plays a causal role in FM, it is essential to assess whether microbiota alterations can induce an FM-like phenotype. To this end, a recent study in Neuron provided the first direct experimental evidence (97). Cai et al. collected fecal microbiota from female FM patients and healthy controls and orally transplanted them into germ-free mice (108). Remarkably, mice transplanted with microbiota from women with FM developed chronic pain behaviors over the next several weeks, including hyperalgesia to mechanical, thermal, and cold stimulation, decreased spontaneous activity, and increased depression-like behavior (97, 108). This abnormal state lasted for at least four months, implying that microbiota-associated pain sensitization was durable (109). Perhaps more impressively, subsequent transplantation with microbiota from healthy mice markedly reduced pain hypersensitivity and partially restored normal behaviors (110). These results suggest that FM-associated microbiota are not only correlated with the disease state, but may substantially contribute to the maintenance of pain—though it is important to note that such animal model findings cannot be directly equated with established causality in the complex, multifactorial context of human disease (111).

To gain insight into potential mechanisms, the study conducted multidimensional analyses in the mice (108). Mice colonized with FM-associated microbiota had elevated numbers of spinal microglia and peripheral inflammatory monocytes, suggesting immune system activation (112). Metabolomic studies also showed changes in the serum amino acid and bile acid concentrations, which were consistent with those reported in the patients (113). Most importantly, the mice had decreased density of epidermal nerve fibers in their skin, mirroring a small-fiber neuropathy seen in some FM patients (114). Overall, these findings strongly suggest that disturbances in gut microbiota can trigger pain and other pathological changes associated with FM (115).

In addition to the FMT model, other animal models have also investigated the microbiota/pain interaction (116). In some mouse studies, for instance, the microbiota has been depleted by antibiotics, followed by transplantation of particular bacterial strains or microbial populations, to examine effects on pain (117). While models specific for FM are lacking, in models of widespread pain or chronic fatigue, depletion of the microbiota typically reduces pain-related responses while supplementation with some probiotics relieves experimental pain (116, 117). These results indicate the microbiota regulates host pain and that certain microbiota may have a pronociceptive or antinociceptive effect (116). Certainly, animal models are not ideal for reproducing the human disease, particularly when there are no clear-cut histological or biochemical markers for FM (35). But the studies by Cai and colleagues partially circumvented this issue through human microbiota transplantation and created a convincing model of causality (116, 117).

### 4.2 Clinical observational studies: evidence of association

Population-based studies have primarily focused on comparing gut microbial differences between patients with FM and healthy controls and correlating these differences with clinical characteristics (118). A series of cross-sectional cohort studies conducted since 2020 in Europe, North America, and Asia have consistently found reduced microbial diversity and altered gut microbial structure in patients with FM (119). For example, Canadian investigators reported that more than 20 bacterial taxa differed significantly in abundance between patients with FM and controls, with increased abundance of certain members of the order Bacteroidales and reduced abundance of some members of the family Ruminococcaceae (28, 76). Similarly, a Scientific Reports study by Lopez de Coca and colleagues in Spain observed significant separation between FM and healthy groups in beta diversity, as measured by Bray–Curtis distance (118).

Some studies have attempted to apply machine learning to distinguish patients with FM from healthy individuals based on gut microbial data, reporting classification accuracies of 80%–90% using the abundance profiles of a limited number of key taxa (28, 119). Of particular interest, increasing numbers of reports have linked symptom severity to microbial signatures (118, 119). For instance, patients with higher pain scores tend to exhibit gut microbial patterns that deviate further from a healthy state; patients with severe anxiety or depression show higher proportions of potentially toxigenic organisms such as enterococci and clostridia; and those with pronounced morning stiffness or fatigue tend to have lower abundance of certain SCFA-producing bacteria (118, 119). Although these findings do not prove causality, they suggest that the gut microbiota may partly explain the heterogeneity of FM symptoms (76, 118, 119).

Clinical observational studies have also explored the influence of environmental and lifestyle factors on the microbiota–FM relationship (120–122). Diet is one of the major external factors shaping the gut microbiota (76, 122). Some investigators have examined associations between dietary patterns and microbial composition in FM and found that patients who favor high-sugar, high-fat diets tend to have lower abundance of anti-inflammatory bacteria and more severe symptoms (76, 118). Conversely, patients consuming diets rich in dietary fiber and polyphenols, resembling a Mediterranean-style pattern, tend to have higher microbial diversity and report better quality of life (121, 122). These observations imply that dietary modification may improve FM-associated microbial abnormalities and thereby alleviate symptoms (32).

### 4.3 Clinical intervention studies: early therapeutic signals

If the gut microbiota plays a meaningful role in FM, microbiota-targeted interventions should, at least in principle, improve patient outcomes (123). In recent years, several small-scale studies have explored this possibility using probiotic supplementation, prebiotic fiber, dietary modification, and fecal microbiota transplantation (FMT) (123, 124). Probiotics and prebiotics, owing to their favorable safety profile, were investigated earlier, whereas FMT has generally been restricted to research settings because of its greater potential risks (125). Although the available evidence remains limited, it provides encouraging preliminary support for the therapeutic value of gut-targeted strategies (123–125).

With respect to probiotics, a representative double-blind randomized controlled trial conducted in Spain in 2018, although outside the main time frame of the present review, was among the first to report the effects of probiotics in FM (126). In that study, 30 patients with FM received either a multispecies probiotic preparation containing eight bacterial strains or placebo daily for eight weeks. Critically, the primary pain outcome did not improve significantly in the probiotic group—a negative result that warrants explicit emphasis, as it runs counter to the hypothesis that probiotics can reduce FM-related pain. The probiotic group did show improvements in impulsivity and decision-making performance and a trend toward reduced depression and anxiety scores, suggesting domain-specific rather than global benefit. In the following years, several additional small randomized controlled or open-label trials further evaluated probiotics in FM (126). For example, a 2022 Italian trial using a formulation containing Bifidobacterium longum and Lactobacillus plantarum found that fatigue scores and sleep quality improved more than with placebo after eight weeks, and circulating inflammatory markers such as TNF-α decreased (127). The inconsistency across probiotic studies likely reflects several sources of heterogeneity: differences in bacterial strains and their dosages, variable treatment durations (ranging from 4 to 12 weeks), and use of divergent primary outcome measures (pain, cognition, mood, or global function). These methodological variations make cross-study comparison difficult and underscore the need for standardized protocols in future trials. Overall, probiotic supplementation may help alleviate comorbid symptoms such as mood disturbance, sleep impairment, and cognitive dysfunction, but evidence of meaningful analgesic benefit remains inconsistent (128).

In terms of dietary intervention, large randomized controlled trials specifically targeting FM are still lacking (99). However, given the high rate of coexistence between IBS and FM, some clinicians have implemented low-FODMAP diets in patients with FM and comorbid IBS (99, 129). These patients frequently report marked improvement in gastrointestinal symptoms, and some also experience reductions in pain and fatigue (129). In addition, a pilot interventional study published in 2023 investigated the effects of a very low-calorie ketogenic diet (VLCKD) in obese women with FM, showing improvements in Fibromyalgia Impact Questionnaire and Hospital Anxiety and Depression Scale scores after the ketogenic phase and after carbohydrate reintroduction (130). A newer 2025 microbiome-oriented dietary study further suggested that a carb-free oloproteic/low-glycemic dietary strategy may modulate bacterial and fungal gut communities in FM, especially with evidence for suppression of Ascomycota/Candida-related signals, but it was not a large randomized placebo-controlled trial and did not support the exact wording that “beneficial methanogenic microbes” were reduced (122). These findings suggest that dietary manipulation may help alleviate some FM symptoms, possibly in part by modifying the intestinal metabolic and microbial environment. Nevertheless, the long-term feasibility and safety of strict dietary interventions remain important considerations (99, 122, 130).

Finally, FMT has emerged as a particularly powerful strategy for restructuring the gut microbiota and has already demonstrated success in metabolic disorders and Clostridioides difficile infection. Its application to FM, a centrally mediated pain syndrome, was initially controversial, but significant progress has emerged since the mid-2020s (97). In addition to the mouse FMT experiments described above, Cai and colleagues conducted an open-label clinical trial in 14 women with severe FM (97). These patients were given five doses of oral freeze-dried fecal microbiota capsules from healthy women (97). There was no placebo control group, as ethical considerations precluded its inclusion. The findings were promising: 12 patients (85.7%) had significant improvement in pain scores following treatment compared to baseline, and several improved by more than 30%, a recognized threshold for clinical significance. Sleep disturbance, fatigue, depression, and anxiety also decreased. Objective measures of pain thresholds (cold pain) increased, suggesting reduced cold hypersensitivity. Fecal microbiome profiling confirmed engraftment of donor microbiota in participants. However, several important limitations must be acknowledged. For subjective pain and functional outcomes, placebo effects can be substantial—particularly in open-label settings where patients know they are receiving active treatment. Without a double-blind, placebo-controlled design, it is not possible to disentangle specific treatment effects from expectation-related improvements. Therefore, while these open-label results are hypothesis-generating, they should be interpreted with caution pending confirmation in rigorously controlled trials (97).

Then, in 2024, a group from Shanghai, China, applied an open randomized controlled design to assess the effectiveness of FMT (125). Fang and co-workers randomly assigned 60 FM patients into either an FMT group that was administered oral fecal capsules or a standard treatment group for three months. While a complete placebo control was not used for ethical reasons, randomization was used. The FMT group had better pain intensity, fatigue, inflammatory marker levels, and gut microbial diversity than the control group (125). This study was published in the Journal of Pain, and it offered higher-level evidence in humans. However, given that the study did not include a placebo and double-blinded design, the results should be viewed with caution (125).

Overall, the existing clinical evidence suggests that approaches that can help improve the gut microbial environment, whether relatively non-invasive methods such as probiotics and diet or more invasive methods such as FMT, may be effective in relieving FM symptoms to varying degrees (101, 109, 123, 125). The results suggest a new direction for FM treatment. But the studies are small and larger, carefully designed studies are required to establish their effectiveness and safety (101, 109, 123, 125).

## 5 Limitations and future perspectives

Despite the growing body of evidence linking the gut microbiota to FM, several important limitations remain (123). First, most available studies are based on relatively small sample sizes, which restricts statistical power and may contribute to inconsistency across findings (109, 123). Second, substantial heterogeneity exists in study design, patient selection, microbiome analysis methods, and clinical outcome measures, making direct comparison between studies difficult (76, 109, 123). Third, most current evidence is cross-sectional in nature and therefore insufficient to clarify the temporal sequence between microbiota alterations and disease onset (76, 123, 131). In addition, long-term follow-up data are scarce, limiting evaluation of the durability and safety of microbiota-targeted interventions (76, 109, 123). A critical and largely underaddressed limitation deserves particular emphasis: the confounding effects of medication use and lifestyle factors on gut microbial composition. Patients with FM commonly use long-term pharmacotherapy, including analgesics (e.g., NSAIDs, opioids), antidepressants (e.g., SSRIs, SNRIs such as duloxetine and milnacipran), and occasionally antibiotics—all of which are well-established modulators of the gut microbiome. Antidepressants in particular have been shown to alter bacterial diversity and specific taxon abundances independently of any underlying disease. Furthermore, pain-related sedentary behavior, disrupted sleep, and dietary preferences common among FM patients (e.g., reduced fiber intake, higher consumption of processed foods) are themselves potent drivers of dysbiosis. The existing literature largely attributes observed microbial alterations to the disease process itself, without adequately controlling for these secondary effects. Future studies must rigorously document and statistically account for medication history, physical activity, diet quality, and sleep patterns in order to distinguish primary disease-associated microbial changes from those that are secondary consequences of pharmacotherapy or lifestyle modification.

Future research should therefore prioritize several directions (76, 109, 119, 123). Large prospective cohort studies are needed to determine whether microbiota alterations precede the development of FM or arise secondarily during disease progression (76, 109, 123). Multi-omics approaches integrating metagenomics, metabolomics, transcriptomics, and immunophenotyping may help identify key pathogenic microbial taxa, metabolites, and host pathways (76, 119),. Well-designed randomized controlled trials are also essential to establish the true efficacy and safety of microbiota-targeted interventions, including probiotics, dietary modification, and FMT (76, 109, 123). Furthermore, greater attention should be paid to individual variability in microbiota composition, symptom patterns, and treatment response, which may ultimately support the development of personalized microbiome-based therapeutic strategies (109, 119, 123).

## 6 Conclusion

An increasingly robust body of evidence supports a close association between rheumatic disease progression and the gut microbiota. In FM, a complex chronic pain syndrome, recent studies suggest that gut microbiota dysbiosis may represent an important yet previously underappreciated component of its pathophysiology. Reduced microbial diversity and altered metabolic function in patients with FM are closely associated with immune activation, central sensitization, and worsening clinical symptoms. Through multiple interconnected pathways—including immune regulation, neuroendocrine signaling, and microbial metabolite production—the gut microbiota may contribute both to disease onset and to the persistence of symptoms in FM. Animal studies further suggest that dysbiotic microbial communities can induce FM-like pain behaviors and neuroimmune alterations; while these findings are consistent with a causal contribution, phenotypic induction in animal models cannot be equated with established causality in the complex, multifactorial context of human disease. Although the precise etiology of FM remains incompletely defined, the gut microbiome offers a novel conceptual framework for understanding the disorder and a promising target for intervention. On this basis, microbiome-based strategies, including probiotic supplementation, dietary modulation, and fecal microbiota transplantation, have been explored in patients with FM and have shown preliminary benefits for pain, fatigue, mood, and related symptoms. These findings suggest that modulating the gut may offer a means of alleviating widespread pain and the multidimensional symptom burden in FM. Nevertheless, the current evidence base remains limited by small sample sizes, methodological heterogeneity, insufficient long-term follow-up, and inadequate control for confounders such as medication use and lifestyle factors. Future research should therefore prioritize large prospective cohorts, multi-omics mechanistic studies, and rigorously designed randomized controlled trials to clarify causality, identify key pathogenic factors, and establish the efficacy and safety of microbiota-targeted therapies. FM is fundamentally a disorder of multifactorial, multisystem dysregulation, and the gut microbiota may represent a critical bridge linking environmental, immune, neural, and metabolic influences. In the era of precision medicine, deeper understanding of this bridge may open new diagnostic and therapeutic possibilities not only for FM but also for other rheumatic diseases, including personalized microbiota-corrective interventions and biomarker-assisted diagnosis. Overall, the role of the gut microbiota in the development of FM warrants continued and intensive investigation. As research advances, it is reasonable to hope that better characterization of gut–brain interactions may ultimately contribute to improved therapeutic strategies that alleviate pain and enhance quality of life for patients with FM.
